# Complement inhibition attenuates acute kidney injury after ischemia-reperfusion and limits progression to renal fibrosis in mice

**DOI:** 10.1371/journal.pone.0183701

**Published:** 2017-08-23

**Authors:** Juan S. Danobeitia, Martynas Ziemelis, Xiaobo Ma, Laura J. Zitur, Tiffany Zens, Peter J. Chlebeck, Edwin S. Van Amersfoort, Luis A. Fernandez

**Affiliations:** 1 Department of Surgery, Division of Transplantation, University of Wisconsin-Madison, Madison, WI, United States of America; 2 Pharming Technologies BV, Leiden, the Netherlands; Universidade de Sao Paulo, BRAZIL

## Abstract

The complement system is an essential component of innate immunity and plays a major role in the pathogenesis of ischemia-reperfusion injury (IRI). In this study, we investigated the impact of human C1-inhibitor (C1INH) on the early inflammatory response to IRI and the subsequent progression to fibrosis in mice. We evaluated structural damage, renal function, acute inflammatory response, progression to fibrosis and overall survival at 90-days post-injury. Animals receiving C1INH prior to reperfusion had a significant improvement in survival rate along with superior renal function when compared to vehicle (PBS) treated counterparts. Pre-treatment with C1INH also prevented acute IL-6, CXCL1 and MCP-1 up-regulation, C5a release, C3b deposition and infiltration by neutrophils and macrophages into renal tissue. This anti-inflammatory effect correlated with a significant reduction in the expression of markers of fibrosis alpha smooth muscle actin, desmin and picrosirius red at 30 and 90 days post-IRI and reduced renal levels of TGF-β1 when compared to untreated controls. Our findings indicate that intravenous delivery of C1INH prior to ischemic injury protects kidneys from inflammatory injury and subsequent progression to fibrosis. We conclude that early complement blockade in the context of IRI constitutes an effective strategy in the prevention of fibrosis after ischemic acute kidney injury.

## Introduction

Acute kidney injury (AKI) is defined as an abrupt and rapid reduction in kidney function resulting in retention of urea and nitrogen waste products along with failure to maintain fluid, electrolyte and acid-base homoeostasis [1]. AKI is usually multifactorial and develops from either obstructive, toxic, infectious and ischemic insults to the kidney such as renal ischemia-reperfusion injury (IRI) which is the most common etiology and an unavoidable consequence of renal transplantation [2]. Following ischemia, restoration of blood flow to ischemic tissue results in the coordinated activation of innate and acquired immune responses that trigger tissue inflammation. This process is characterized by rapid immunocyte mobilization, up-regulation and expression of cell-adhesion molecules and an influx of activated polymorphonuclear (PMN) and mononuclear cells into the site of injury [3, 4]. In addition, pro-inflammatory cytokines are also expressed along with reactive oxygen species (ROS), known to potentiate tissue damage and immunogenicity [2]. The objective of the inflammatory process is to protect the local environment from noxious stimuli and to repair the lost tissue components. Infiltrating macrophages in the injured tissue secrete pro-fibrogenic cytokines such as transforming growth factor-β1 (TGF-β1) which is a potent chemoattractant for cells of macrophage-monocytic lineage and stimulates myofibroblast transformation via epithelial to mesenchymal transition (EMT) [5]. Macrophages, tubular epithelial cells, and myofibroblasts have all been shown to synthesize TGF-β in the context of acute and chronic renal injury and organized activation of these cells results in collagen formation, extracellular matrix deposition and ultimately, renal fibrosis in experimental animal models [6].

The complement system is a major regulator of the inflammatory response [7]. It is composed of circulating and locally synthesized proteins that are activated in an organized manner upon stimulation by infectious agents or tissue injury. Three distinct activation pathways (classical, lectin and alternative) have been described and all converge in the assembly of the membrane attack complex (MAC), the terminal product critical for defense against pathogens and which has been also implicated in the mechanisms of tissue injury during inflammation [8]. In addition, activation of the complement cascade results in the cleavage of components C3 and C5 and the generation of anaphylatoxins (C3a and C5a), known potent amplifiers of the inflammatory response, and the release of opsonins such as C3b which are important mediators in the process of antigen presentation [9, 10].

Multiple animal studies have shown a pivotal role for complement in renal IRI [8]. In mice, local synthesis of C3 formation at the level of the tubular epithelium followed by MAC deposition has been shown to induce complement-mediated tubular cell injury after renal IRI [11]. In addition, C3a and C5a release are widely associated with kidney damage via recruitment and activation of innate immunocytes to the site of injury which results in ROS formation, cell necrosis and apoptosis [12, 13]. More importantly, direct C5a interaction with the C5a receptor (C5aR) on parenchymal cells, independent of neutrophil activation, has been shown to mediate tubular injury in the context of IRI [14]. Although all three pathways of activation have been implicated in the mechanisms of inflammatory tissue injury, recent data suggest a predominant role for the mannose-binding lectin (MBL) pathway mediating renal, neural, myocardial and intestinal IRI [15–18]. Furthermore, simultaneous blockade of both the classical and lectin pathways has been shown to confer a protective effect in the context of renal ischemia reperfusion injury [19].

Complement inhibitors have been successfully used in small and large animal models of ischemia reperfusion injury in in the past decade [19–22]. Specifically, a monoclonal antibody that binds the C5 fragment (Eculizumab) which prevents cleavage and C5a formation and inhibits terminal complement activation and recombinant inhibitors of C1 fraction (C1INH) which prevents activation of classical and lectin pathways of complement activation have shown protective effects in renal ischemia reperfusion injury and show promise in the management of acute and chronic rejection after renal transplantation [23, 24]. However, the role of complement in the progression to renal fibrosis after ischemic injury is incompletely understood and currently there are no effective anti-fibrotic therapies to prevent the progression to fibrosis after ischemic insult to the kidney. We hypothesized that complement blockade by intravenous delivery of C1INH prior to the onset of injury would potentially decrease the inflammatory response to ischemia and reduce the progression to fibrosis in a model of unilateral renal IRI. Our results indicate that pre-treatment with C1INH attenuates inflammatory injury, prevents neutrophil and macrophage migration/activation and ultimately limits the progression to fibrosis by decreasing TGF-β1 signaling and collagen deposition at the site of injury.

## Concise methods

### Experimental animals

Male C57/B6 mice weighing between 20–25 g obtained from the Jackson Laboratories (Bar Harbor, ME) were used in all experiments. Animals had free access to food and water and were housed and maintained at the University of Wisconsin-Madison Laboratory Animal Resource facility in accordance with Institutional Animal Care and Use Committee and NIH guidelines for appropriate care and use of laboratory animals. Animals were assessed daily by veterinary staff at our institution and by qualified investigators in our group. Criteria used for termination of an experiment and euthanasia were as following: sustained signs of dehydration that do not respond to fluid administration, extreme lethargy for 2 or more consecutive days, extreme pain and/or discomfort that does not respond to administration of analgesics, suspected internal bleeding, signs of systemic infection, behavioral changes, abnormal appearance, ruffled coat, hunching, extreme weight loss suggestive of inadequate post-operative recovery. The University of Wisconsin-Madison Institutional Animal Care and Use Committee approved all animal research presented in this manuscript.

### Experimental design

**Fig 1:**
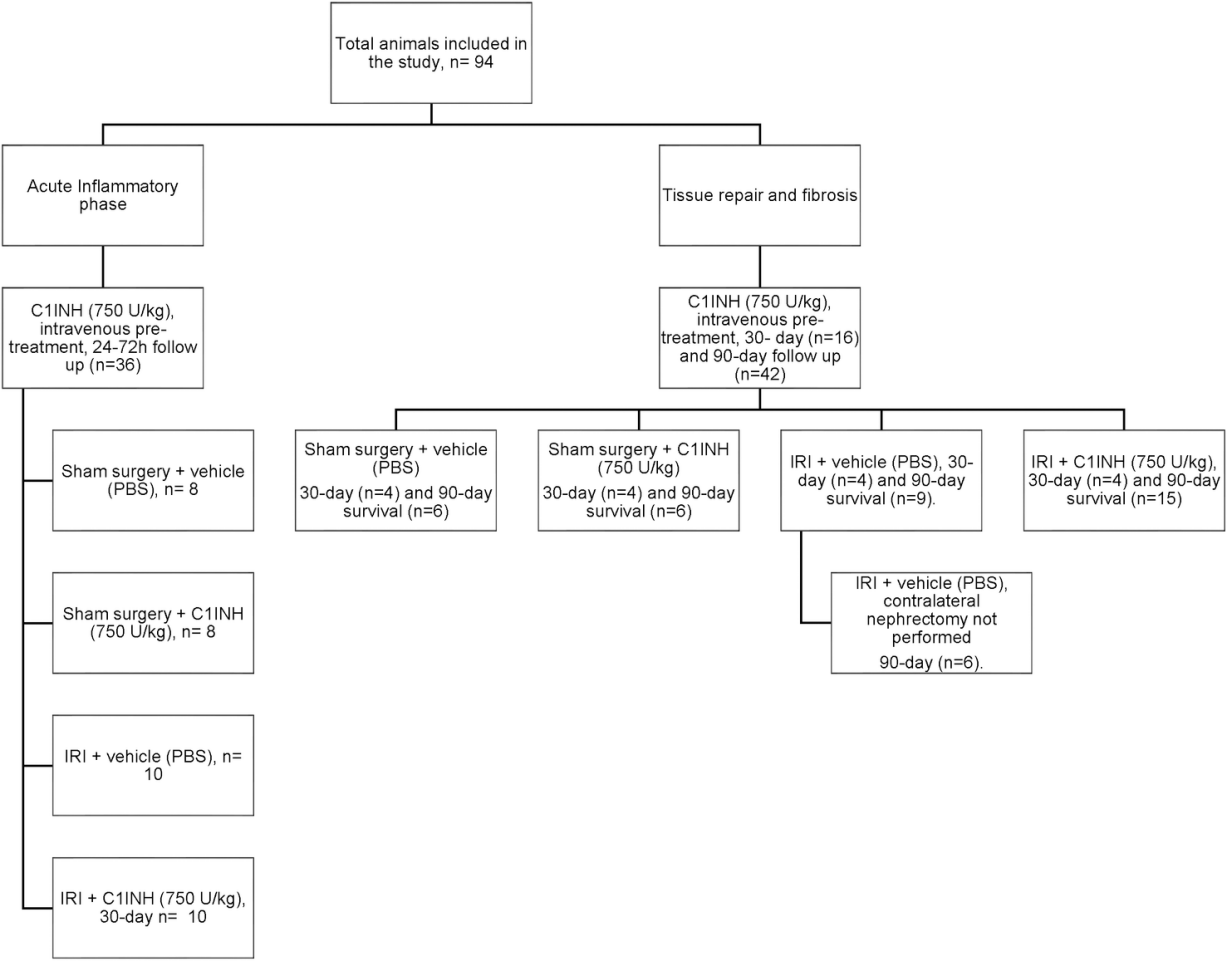
Diagram of the experimental design used for the study.

We divided the study into four experimental groups: Sham operated animals receiving phosphate buffered saline (PBS) (Sham + PBS), sham animal receiving intravenous recombinant human C1-inhibitor (C1INH) (Sham + C1INH), mice subjected to IRI receiving PBS (IRI + PBS) and mice subjected to IRI receiving C1INH (IRI + C1INH). Assessments were performed at days 1, 3, 7, 30 and 90 after reperfusion (Fig 1). Sterile PBS (0.5 ml) and C1INH (750 U/kg) were given intravenously via tail vein injection 1 hour prior to surgery. C1INH (Ruconest) was kindly provided by Pharming Technologies BV (Leiden, The Netherlands). C1INH has been widely studied in mouse models of IRI [22, 25] and we used a dose of 750 U/kg per mouse. Appropriate dosing ranges for this study were determined by the manufacturer based on data obtained during the development and preliminary testing of the drug.

### Ischemia-reperfusion injury model

Briefly, experimental animals were anesthetized with isoflurane (1–2%) and placed on a heating pad to maintain body temperature (37–39°C). Under aseptic conditions, a midline incision was performed and the right renal pedicle identified, ligated and a right nephrectomy performed. The left renal pedicle was then identified and clamped using a small non-traumatic clamp. After visual confirmation of ischemic changes, the kidney was returned to the peritoneal cavity and vessel occlusion was maintained for a 60-minute period. The clamps were then released and blood flow restoration confirmed visually. Incision was closed in two layers using 4–0 absorbable suture and animals were volume resuscitated using normal saline (0.5–1.0 ml) injected subcutaneously. Subcutaneous buprenorphine (0.05 mg/kg) was given every 8 hours to ensure post-operative analgesia. To study acute inflammatory responses after IRI we allowed reperfusion for 24h and 72h post-injury. To study post-ischemic progression to fibrosis animals were followed for 30 and 90 days after initial injury. Since most animals in the IRI + PBS group did not survive past 7 days, a subgroup of mice (n = 6) were allowed to retain the contralateral native kidney to ensure 30 and 90-day survival and data from these experiments excluded from survival analysis. Following the reperfusion phase animals were anesthetized, a second laparotomy performed followed by left nephrectomy and blood was obtained by cardiac puncture. Animals were euthanized by exsanguination under anesthesia following institutional guidelines. Sham animals were anesthetized and a midline incision performed, renal pedicles dissected but not clamped. They were maintained for 60 minutes and the incision was then closed in two layers. Kidneys were then recovered at the same time points as experimental animals. Native kidneys were obtained from healthy anesthetized animals and processed immediately.

### Renal function

Serum was collected at 1, 3, 7 and 90 days post-injury and creatinine levels measured using a VetTest Chemistry Analyzer (Idexx laboratories, Westbrook, ME). Values obtained at 30 and 90 days are reported in the supplementary material (S1 Fig). Animals in the IRI-PBS group were not subjected to contralateral nephrectomy and data are not directly comparable among all groups.

### Histopathology and immunofluorescence

Renal tissue was fixed in 10% formaldehyde, embedded in paraffin, and mounted onto slides for staining. Hematoxylin/Eosin stains were used to evaluate renal tubular injury scores. Briefly, random fields were semiquantitatively evaluated separately by two blinded investigators (×200 magnification) with the following scoring system: 0 = no injury; 1 = 1–25% of area injured; 2 = 25–50%; 3 = 51–75%; and 4 = 76–100%. Immunolabeled antibodies for F4/80 (clone BM8, eBioscience), Ly6G (clone 1A8, Biolegend), complement component C3/C3b (Abcam), alpha smooth muscle actin (clone EPR5368, Abcam-Epitomics) and Desmin (Abcam-Epitomics). For collagen staining slides were deparaffinized and immersed in picrosirius red (Sigma-Aldrich) solution for 1 hour, dehydrated in 100% ETOH and cleared in xylene. Images were analyzed blindly by two separate investigators and quantification was performed using ImageJ software (NIH, Bethesda, MD). Positive-staining area and cell counts for each image were quantified using color-separation and background-subtraction, automatic thresholding and particle-analysis algorithms as previously published [26].

### Western blot analysis

Total protein was extracted from frozen kidney samples, solubilized in mammalian protein extraction reagent (Thermo Scientific, Waltham, MA), separated by SDS-page on 4–20% gels (Biorad) and transferred onto a nitrocellulose membrane. We used monoclonal rat anti-mouse C5a antibody (RD systems) and the phosphorylated and non-phosphorylated forms of ERK1/2 were detected using rabbit anti-mouse p44/42 MAPK polyclonal antibody (Cell Signaling). Rabbit polyclonal to TGF beta-1 (Abcam) and rabbit anti-mouse polyclonal beta-tubulin antibody (Thermo Scientific). Densitometric analysis was performed using ImageJ software and samples were normalized to density of the endogenous control.

### Circulating cytokine measurement

Plasma concentration of circulating cytokines IL-6, MCP-1 (CCL2), IL-10 and KC (CXCL1) was determined by ELISA according to manufacturers’ instructions (R&D Systems).

### Gene expression

Total kidney RNA was extracted from snap-frozen tissue using the Qiagen RNeasy Kit (Qiagen, Germantown, MD) following instructions and including on-column DNase digestion step. RNA (0.5 μg) was reverse transcribed using Omniscript Reverse Transcription kit (Qiagen) following manufacturer’s instructions. Quantitative PCR was performed using Taqman Universal PCR Master Mix and a Taqman gene expression assays (Life Technologies, Carlsbad, CA) with mouse gene specific primers for IL-6 (mm00446190_m1), MCP-1 (mm00436450_m1), bradykinin receptor 1 (mm04207315_s1), bradykinin receptor 2 (mm00437788_s1), CXCL1 (mm04207460_m1), IL-4 (mm00445259_m1), IL-13 (mm00434204_m1), IL-10 (mm01288386_m1) and the endogenous control GAPDH (mm99999916_g1) (Life Technologies, Carlsbad, CA) on a GeneAmp 5700 Fast Sequence Detection System (Life Technologies, Carlsbad, CA). Fold change was calculated using the ^ΔΔ^Ct method relative to untreated (native) mouse and GAPDH as the endogenous control.

### Statistical analysis

Comparisons between multiple groups was performed by one-way analysis of variance and Bonferroni’s post-test correction for post hoc pairwise between-group comparisons. Kruskal-Wallis test and Dunn’s post-test correction were applied to data sets with non-normal distribution. Survival analysis was performed with Kaplan–Meier survival analysis using the log-rank test. Statistical analyses were performed using GraphPad Prism V5.0. All data are shown as mean ± standard deviation from the mean (SD). The level of statistical significance was defined as a P < 0.05.

## Results

### Intravenous delivery of C1INH protected mouse kidneys from acute ischemia-reperfusion injury.

**Fig 2:**
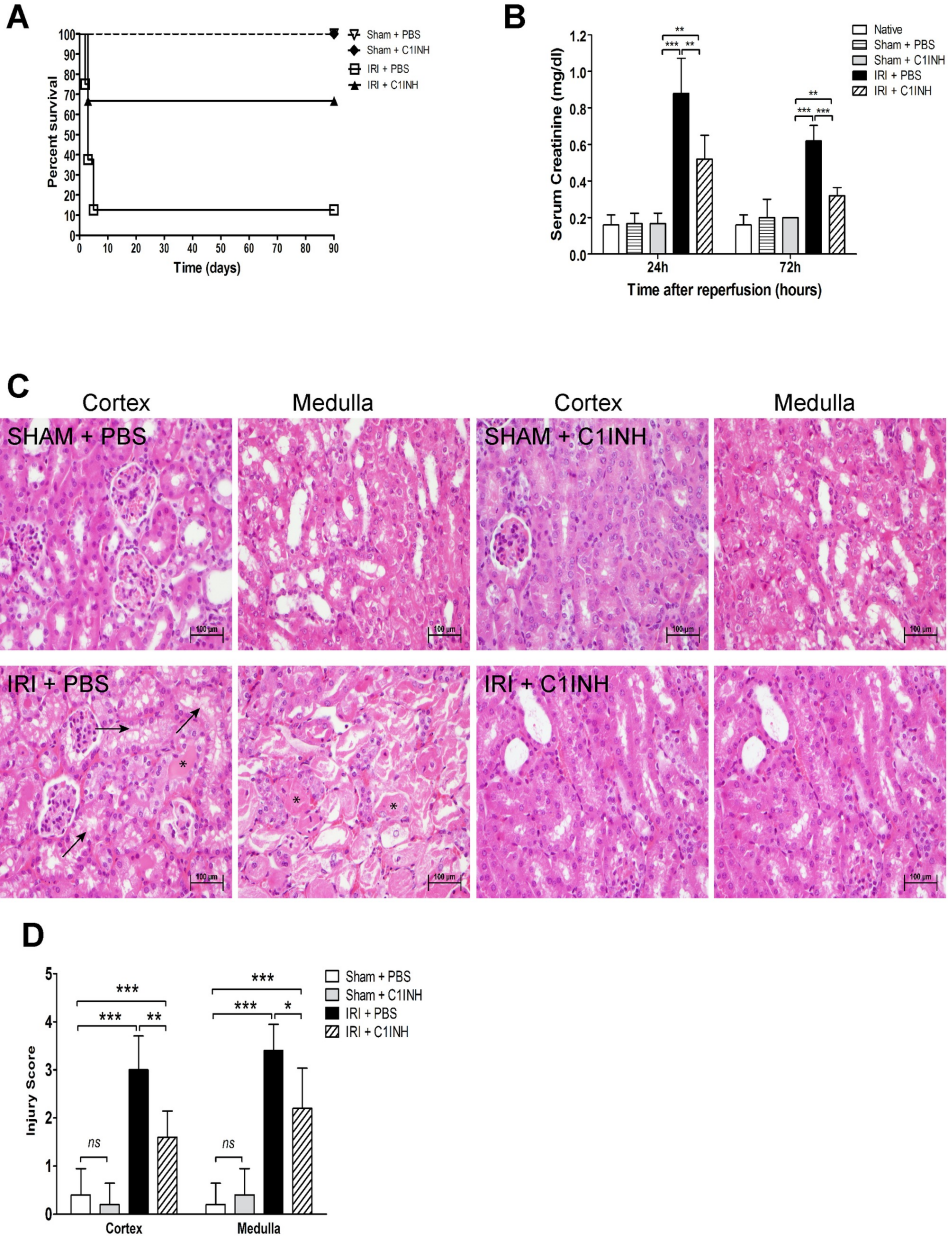
Protective renal effect of complement blockade after ischemia-reperfusion injury. Mice were assigned to four groups: 1) Sham + PBS (n = 6), 2) Sham + C1INH (n = 6), 3) IRI + PBS (n = 9), IRI + C1INH (n = 15). Ischemia was induced by clamping of the renal hilum for 60 minutes and followed by contra-lateral nephrectomy. Sterile PBS or C1INH (750 U/kg) were given intravenously via tail vein injection 1 hour prior to surgery. Serum was collected at 24 and 72 hours after injury and survival was monitored for 90 days after reperfusion. (A) Pharmacological targeting of complement activation using C1INH significantly improves animal survival after IRI (Log-rank test, *P* = 0.0015). (B) Serum creatinine level at 24 and 72 hours after IRI. Groups: 1) Sham + PBS (n = 8), 2) Sham + C1INH (n = 8), 3) IRI + PBS (n = 10), IRI + C1INH (n = 10). (C) Histopathological analysis of kidneys at 24 hours after surgery. Representative light microscopy images of hematoxylin-eosin (H&E) staining of the cortex and medulla of kidneys (200X magnification). Arrows indicate necrotic tubules, and asterisks indicate tubular casts. (D) Renal tubular injury scores (0–4, arbitrary units). All data presented are mean ± SD. Survival data was analyzed by the Kaplan–Meier survival method and the log-rank test. Statistical comparison for creatinine values and tubular injury scoring was performed by one-way ANOVA followed by Bonferroni’s post-hoc correction. *p<0.05, **p<0.01, ***p<0.001.

To determine whether complement blockade using C1INH would impact kidney function following severe IRI, we monitored renal function and survival for a 90-day period. Sham animals and mice subjected to renal IRI of the left kidney underwent contralateral nephrectomy and were allowed to recover post-operatively. Overall survival up to the 90-day endpoint was achieved by 66.6% (10/15) animals receiving C1INH compared to 11.1% (1/9) of PBS treated littermates (p < 0.01). All sham animals receiving either PBS or C1INH survived the 90-day experimental period (Fig 2A). We also monitored renal function at 24 and 72-hours post-reperfusion and documented a significant reduction in serum creatinine levels in animals receiving C1INH compared to PBS treated mice and sham controls (Fig 2B). To assess renal cyto-architecture and tubular injury we performed histologic analysis of kidneys recovered at 72 hours post-IRI. Mice receiving pretreatment with PBS showed widespread vacuolization, tubular dilation, epithelial flattening and abundant cast formation most evident at the level of the renal medulla (Fig 2C). In contrast, kidneys from mice treated with C1INH prior to injury showed conserved tubular morphology, minimal vacuolization and considerably less cast formation in both the cortex and medulla of reperfused kidneys leading to a significantly lower tubular injury score (Fig 2D).

### Pre-treatment with C1INH prior to renal IRI reduced complement activation in the kidney as tested by C5a expression and C3b deposition

**Fig 3:**
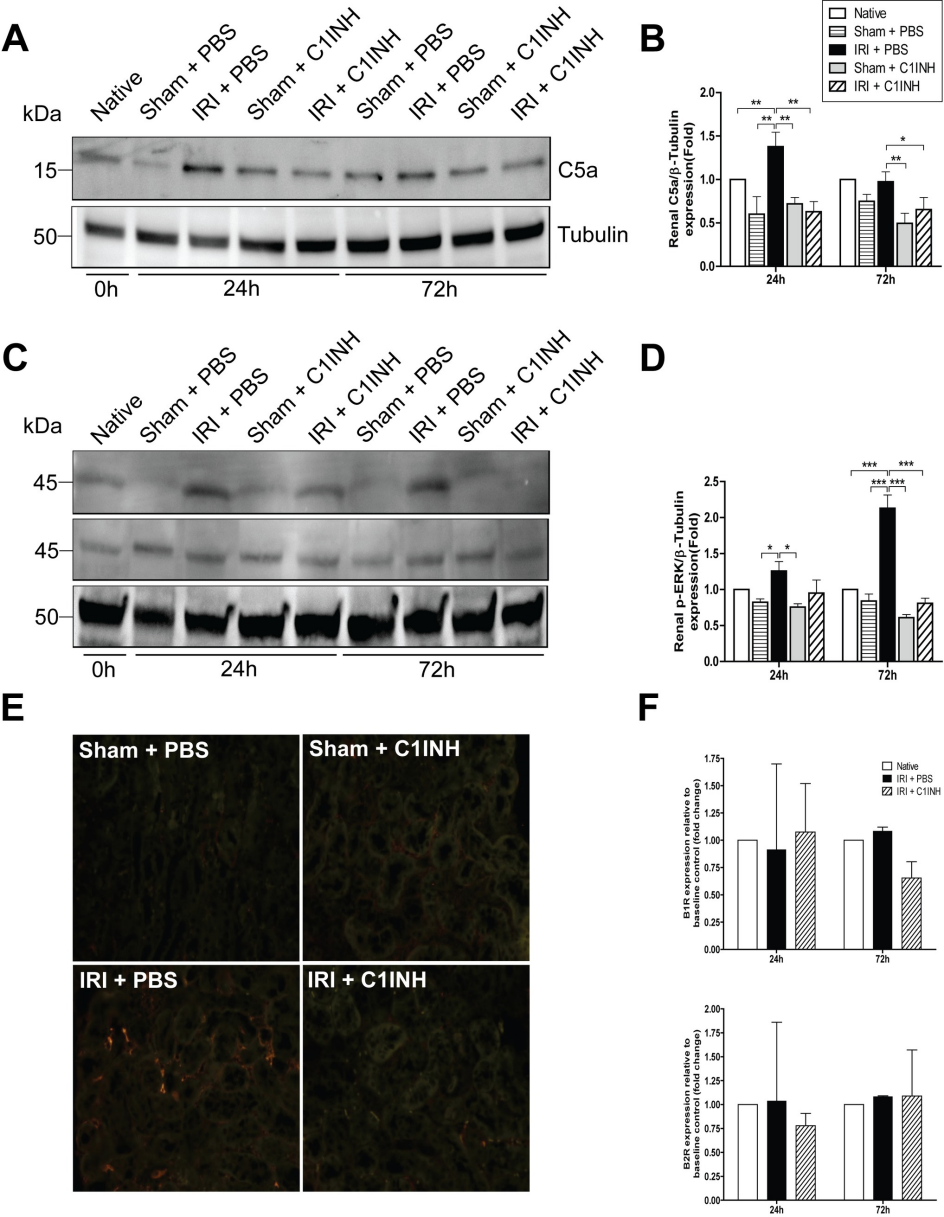
C1INH significantly decreased renal C5a cleavage and limited C3b deposition in kidneys subjected to IRI. (A) Immunoblotting analysis for C5a in renal tissue at 24 and 72 hours post-IRI. (B) Quantification of C5a band density from western blots normalized to β-tubulin. Results are expressed as mean ± SD fold changes (n = 3 for each group) compared with native controls. (C) Immunoblotting analysis of phosphorylated ERK1/2 normalized to total ERK1/2 (p44/42 MAPK) in renal tissue at 24 and 72 hours post-IRI in renal tissue. (D) Quantification of phosphorylated ERK band density from western blots normalized total ERK and β-tubulin. Results are expressed as mean ± SD fold changes (n = 3 for each group) compared with native controls. (E) Representative images of immunofluorescent microscopy performed on renal tissue at 72 hours post-injury targeting C3b deposition (200X). (F) mRNA expression of bradykinin 1 (BR1) and BR2 receptor in renal tissue. Expression was normalized to baseline expression of native controls and GAPDH was used as the endogenous control. Results are expressed as mean ± SD fold changes (n = 3 for each group).

Complement activation, C5 cleavage and release of fraction C5a have been widely implicated in the inflammatory response to ischemia-reperfusion injury [14]. By western blot we measured C5a expression level in kidney samples from animals subjected to IRI at 24 and 72h and used sham animals and native kidneys to establish baseline control levels. At both 24 and 72h we observed a marked increase in C5a expression in kidneys from PBS treated mice when compared to animals receiving C1INH prior to injury (Fig 3A and 3B). Furthermore, we measured the level of phosphorylated ERK1/2 (p-ERK1/2), an important downstream effector of C5aR signaling and documented increased phosphorylation in kidneys from PBS treated mice after IRI at 24 and 72h, and a significant reduction in phosphorylation levels in renal tissue recovered from C1INH treated animals at these time points (Fig 3C and 3D). We also evaluated renal C3b deposition, a marker of complement activation commonly associated with renal IRI [27], and observed a significantly decreased deposition in tissue from C1INH treated mice at 72h when compared to PBS treated littermates (Fig 3E). Lastly, we tested whether IRI induced activation of the contact pathway of coagulation by measuring mRNA expression of the bradykinin receptor 1 and 2 (BR1 and BR2, respectively) as previously described [28]. We did not detect differences in gene expression level in any of the tested groups suggesting the contact pathway was not activated in our model (Fig 3F).

### Complement blockade attenuates the expression of renal pro-inflammatory cytokines and chemokines in the first 72 hours after ischemic injury.

**Fig 4:**
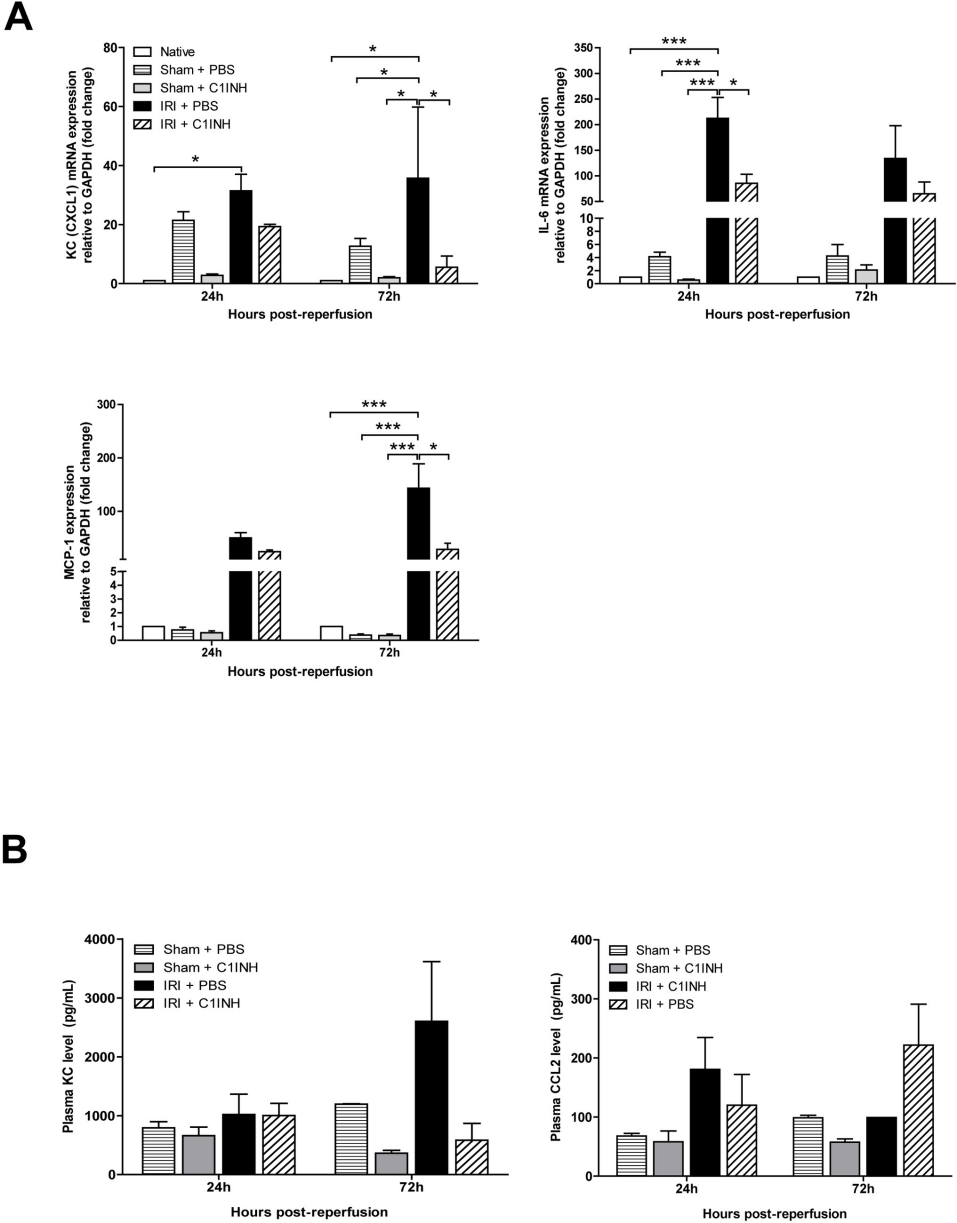
Renal mRNA expression and circulating level of inflammatory cytokines and chemokines at 24 and 72-hours post-injury. RT-PCR analysis was performed on renal tissue recovered at the specified time points from mice in all groups (A) mRNA expression of monocyte chemoattractant protein-1 (MCP-1, n = 6), Interleukin-6 (IL-6, n = 6) and CXCL1 (KC, n = 4). Expression was normalized to baseline expression of native controls and GAPDH was used as the endogenous control. (B) Plasma KC and CCL2 levels post-injury. Cytokine levels determined by ELISA on plasma recovered at the specified time points from mice in all groups (n = 3 for each group). Data are mean ± SD. Statistical comparison was performed by Kruskal-Wallis and Dunn’s post-hoc correction. *p<0.05, **p<0.01, ***p<0.001.

IRI is commonly associated with renal inflammation and early expression of inflammatory cytokines and chemokines. We assessed the expression of IL-6, MCP-1 (CCL2) and CXCL1 by RT-PCR in kidney tissue in the early stages of inflammation and documented an over 200-fold increase in IL-6 mRNA levels at 24h, 140-fold up-regulation in MCP-1 levels at 72h and a 35 fold up-regulation of in CXCL1 mRNA transcripts in PBS treated mice when compared to native controls. Pre-treatment with C1INH significantly reduced the expression of both IL-6 at 24h and MCP-1 and CXCL1 at 72 hours compared to PBS mice (p<0.05) (Fig 4A). We also measured the level of plasma pro-inflammatory cytokines KC, CCL2 but did not observe statistically significant differences between the tested analytes at 24 and 72 hours post-injury (Fig 4B). We could not detect circulating IL-6 or IL-10 in our experiments.

### Pre-treatment with C1INH modulated inflammatory leukocyte recruitment to the site of injury

**Fig 5:**
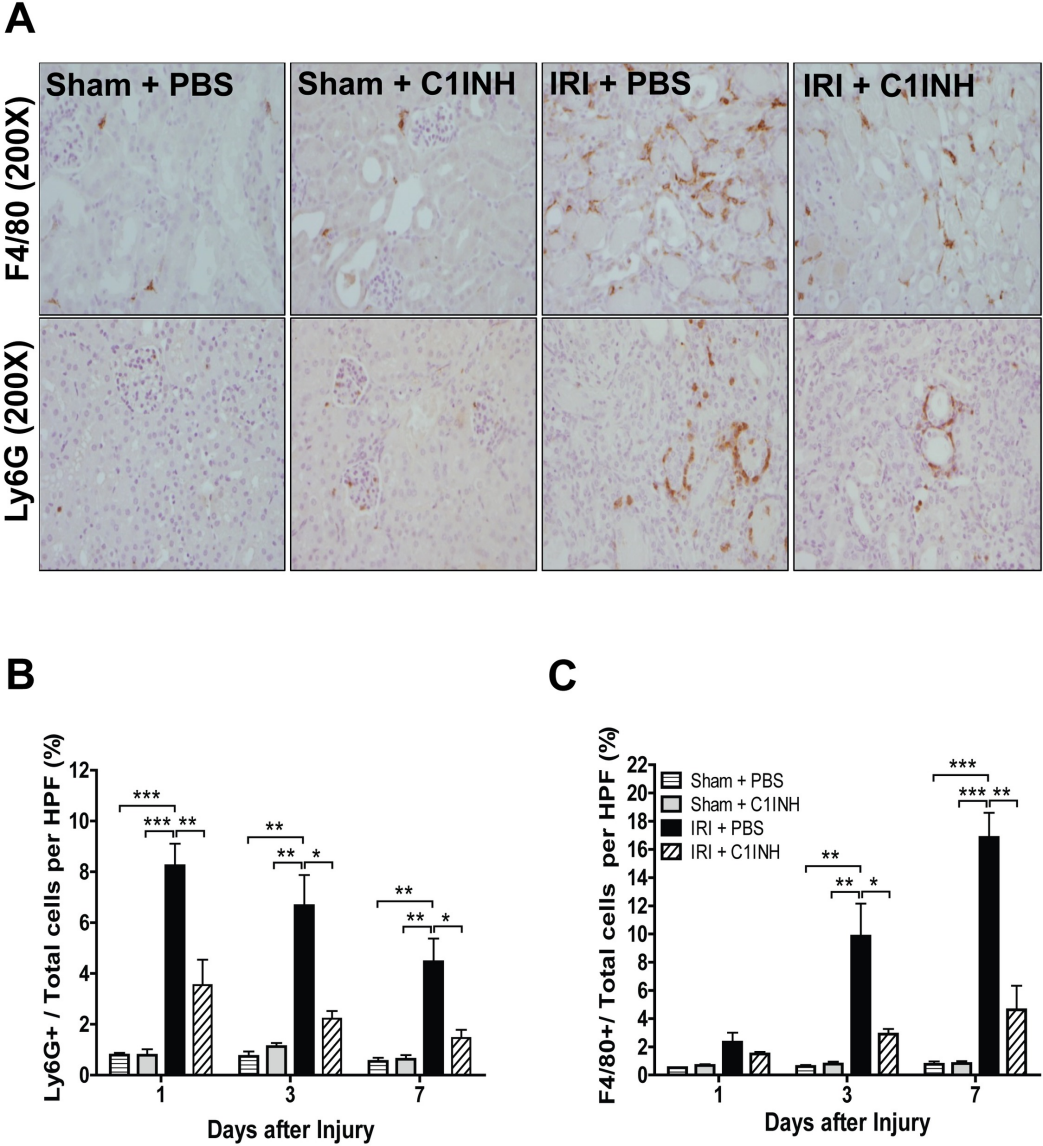
C1INH pretreatment reduces innate immune cell infiltration in post-ischemic kidneys. (A) Immunohistochemical staining for Ly6G (neutrophils) and F4/80 (monocytes/macrophages) in kidney sections obtained from sham animals and mice subjected to IRI in all groups. Kidneys were recovered at 1, 3 and 7 days after ischemic insult. Representative light microscopy images (200X magnification) from each group (n = 6) are depicted. (B) Semi-automated quantification of Ly6G(+) and F4/80(+) cells in each HPF (n = 6 per group). (C) Cell numbers are shown as a percentage relative to total cells per field. Data are mean ± SD. Statistical comparison was performed by one-way ANOVA followed by Bonferroni’s post-hoc correction. *p<0.05, **p<0.01, ***p<0.001.

We analyzed the impact of C1INH therapy in the recruitment of neutrophils (Ly6G+) and macrophages (F4/80+) to renal tissue at 1, 3 and 7 days post-injury. Infiltration by neutrophils was maximal at 24h in mice treated with PBS after IRI and decreased progressively in the first week post injury (Fig 5A and 5B). In contrast, macrophage levels were not different between groups at 24h but gradually increased peaking 7 days after surgery (Fig 5A and 5C). Interestingly, mice receiving C1INH treatment demonstrated limited infiltration of both macrophages and neutrophils and a significant reduction in cellular counts at all evaluated time points when compared to PBS treated mice (p < 0.05).

### Complement blockade attenuated progression to renal fibrosis

**Fig 6:**
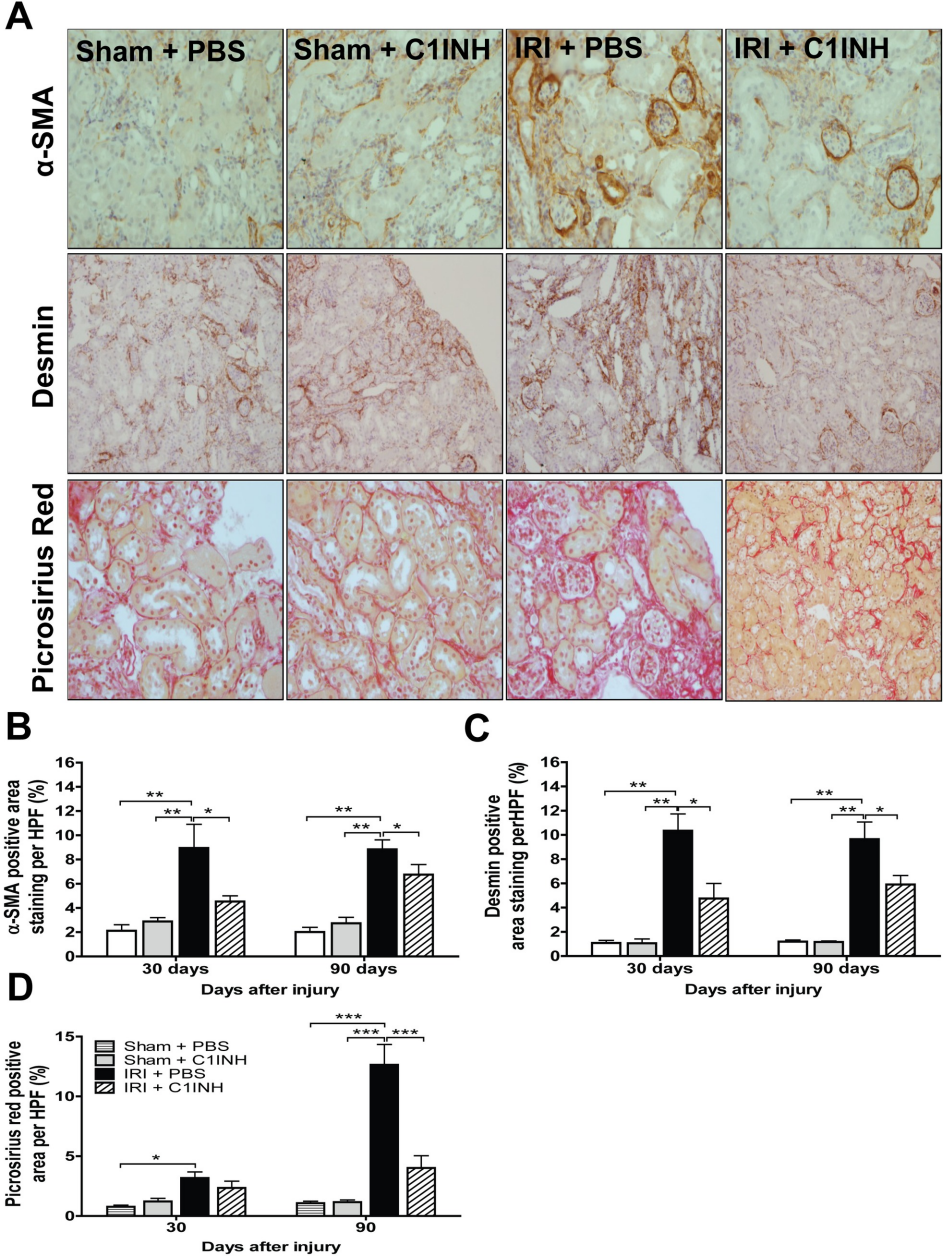
Treatment with C1INH prevented the progression to fibrosis in kidneys subjected to ischemia-reperfusion injury. (A) Immunohistochemical staining for alpha smooth muscle actin (α-SMA), desmin and picrosirius red staining in renal tissue recovered at 30 and 90-days post-ischemic injury from sham + PBS (n = 6), sham + C1INH (n = 6), IRI + PBS (n = 6) and IRI + C1INH (n = 6) mice. Representative light microscopy images (200X magnification) from each group are depicted. Semi-automated quantification of (B) α-SMA(+), (C) Desmin(+) and (D) Picrosirius Red(+) area per HPF. Stained area is calculated and shown as a percentage relative to total tissue area per field. Data are mean ± SD. Statistical comparison was performed by one-way ANOVA followed by Bonferroni’s post-hoc correction. *p<0.05, **p<0.01, ***p<0.001.

**Fig 7:**
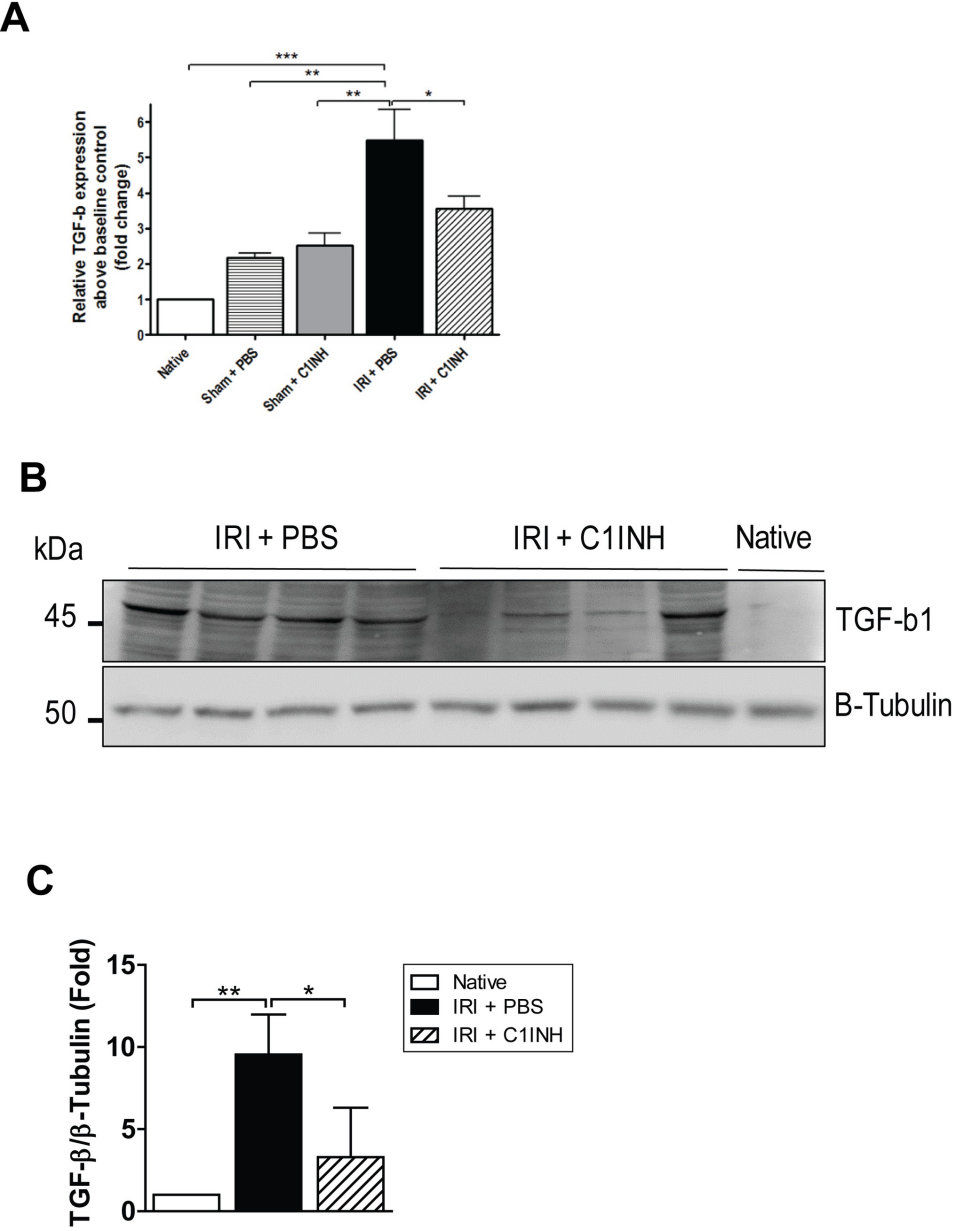
TGF-β1 expression was reduced at 90 days in kidneys recovered from C1INH treated mice. (A) mRNA expression of TGF-β1 in renal tissue at 90-days post-injury. Expression was normalized to baseline expression of native controls and GAPDH was used as the endogenous control. (B) Immunoblotting analysis for TGF-β1 in renal tissue at 90 days post-IRI. (C) Quantification of TGF-β1 band density from western blots normalized to β-tubulin. Results are expressed as mean ± SD fold changes (n = 4 for each group) compared with native controls (n = 1). Data are mean ± SD. Statistical comparison was performed by Kruskal-Wallis and Dunn’s post-hoc correction. *p<0.05, **p<0.01, ***p<0.001.

Acute kidney injury is followed by tissue repair characterized by collagen deposition and fibrosis which ultimately results in progression to chronic kidney disease [29]. To determine if C1INH treatment modified the natural progression to fibrosis, we modified our IRI protocol and allowed a subgroup of mice in the IRI + PBS group (n = 6) to retain the contralateral native kidney to ensure 30 and 90-day survival. We then examined renal sections for α-SMA, desmin and collagen (Fig 6A) staining at these time points and compared them with those from C1INH treated mice and sham operated controls. Staining for α-SMA, desmin and collagen in kidneys from mice in the PBS group was significantly increased. In contrast, we observed a marked attenuation in staining level in kidneys from pre-treated animals at 90-days post-reperfusion (Fig 6B–6D). Since TGF-β is a well-known modulator of fibrosis in multiple models of chronic tissue injury, we evaluated mRNA expression and protein levels of TGF-β1 in renal tissue from mice in all groups. TGF-β1 expression was significant up-regulated at the gene and protein level in kidneys from animals in the PBS group. More importantly, renal tissue from mice pretreated with C1INH evidenced a significantly lower level of expression of TGF-β1 and these results parallel the degree of renal functional impairment and inflammation (Fig 7A–7C).

## Discussion

Considerable attention has been devoted to the pathogenesis of ischemia-reperfusion and the molecular mechanisms that regulate the inflammatory injury in ischemic tissue. Many prevalent conditions such as coronary artery disease, ischemic stroke, trauma, sepsis, and solid organ transplantation are characterized by a combination of direct injury resulting from warm and/or cold ischemia and indirect injury from the dysregulated inflammatory response that ensues [3, 30]. In recent years, both complement and the coagulation systems have recently been shown to play determinant roles in the mechanisms leading to organ damage after IRI [8].

In this study we targeted the complement system by using C1-inhibitor, a plasma serine protease inhibitor known to inactivate proteases of the complement system [31] and observed a significant increase in overall animal survival alongside a marked improvement in renal function and limited progression to fibrosis after severe renal injury. Human C1-inhibitor has been used successfully in rodent models of ischemic injury in the past and several groups have reported similar protective effects following cardiac, neurological and intestinal IRI [20–22, 32, 33]. The mechanisms of protection behind C1INH therapy appear to be diverse. Primarily, MAC (C5b-9) formation has been shown to injure renal epithelial tubular cells in culture independent of immune cell presence and activation in a model of IRI [11]. In addition, C3a and C5a anaphylatoxins play pivotal roles in the modulation of innate and adaptive immunity following injury [12, 34]. In our model, we documented a marked increase in C5a expression at days 1 and 3 post-reperfusion and observed a reduction in cleaved C5a after IRI in mice pretreated with C1INH. This suppressive effect on C5a formation after C1INH therapy has been previously documented by Nürnberger and colleagues and suggests upstream control of the cascade at the level of C1q and MASP1/2, limiting complement activation through the classical, lectin and possibly the alternative pathway as well [35]. The alternative pathway has been described as the most common mechanism of complement mediated renal injury after IRI in rodents and multiple reports have suggested a role for C1INH in regulating the activation of the alternative pathway by preventing binding of C3b to factor B [36]. In our study we observed a decrease in C3b deposition in injured kidneys pre-treated with C1INH in comparison to those receiving PBS only, suggesting that the alternative pathway was also inhibited by the experimental treatment and in agreement with previous reports showing that C1INH can regulate complement activation via the alternative pathway [37]. Complement is a known inducer of pro-inflammatory cytokine and chemokine release and multiple studies have demonstrated the association between elevated IL-6 levels and enhanced renal dysfunction after IRI [38]. In this study we observed a significant down-regulation of renal IL-6, CXCL1 and MCP-1 mRNA levels in response to C1INH pretreatment when compared to untreated controls. This reduction in cytokine expression is not only suggestive of a potent anti-inflammatory effect by C1INH, but also indicates that direct reduction in IL-6 could be partly responsible for the protective effect of therapy. Mice deficient in IL-6 signaling are resistant to chemically induced renal injury and IL-6 blockade has been shown to ameliorate renal function in models of acute kidney injury [39]. In addition, a recent study by Riedemann et. al suggested that IL-6 blockade down-regulated C5aR receptor expression and significantly improved survival in a rodent model of sepsis [40]. This simultaneous reduction of both C5a and IL-6 signaling by C1INH may partly explain the synergistic protective effect and amelioration of tissue function observed in the context of renal IRI.

The trigger for complement activation in sterile injury also remains uncertain. Cell necrosis and apoptosis are hallmarks of ischemia reperfusion injury and release of danger signals (e.g, HMGB1, HSP and S100 proteins) following ischemic insult is a well-known event after ischemic tissue injury [41]. Although current lines of research suggests a predominant role for TLR as targets to prevent post-ischemic inflammatory injury, emerging evidence indicates that complement may also play an important role in the pathogenesis of IRI and crosstalk between both systems is likely to determine the magnitude of the inflammatory response [42]. The contact system, a group of plasma proteins composed of three serine proteinases (coagulation factors XII and XI, plasma prekallikrein and high molecular weight kininogen) are closely related to complement and can also be activated in the setting of renal ischemia reperfusion injury [43]. Several reports have demonstrated up-regulation of both bradykinin receptors (BR1 and BR2) in response to tissue injury and their stimulation activates endothelial NO synthase in the vascular endothelium proteins [44]. In a recent study of renal IRI using BR-null mice, absence of B1R and B2R signaling (and to a lesser extent, the absence of B2R alone) was associated to DNA damage, mitochondrial DNA deletions, apoptosis, and the expression of TGF-β1 [28]. Despite convincing evidence of a role for the contact system in the pathogenesis of renal IRI, we did not detect changes in the expression level of the bradykinin receptors in renal tissue indicating that the contact system was not activated in our model and that the protective effect of C1INH was independent of contact system activity. As mentioned before, C1INH is a versatile molecule with multiple targets. Although we did not observe a significant impact of C1INH therapy on the activation of the contact system by gene expression we did not fully investigate the role of C1INH in fibrinolysis or its impact on coagulation and a more detailed study of the effects of complement inhibition on hemostasis in the context or IRI is warranted.

Tissue infiltration by activated neutrophils and macrophages is a known consequence of reperfusion injury and is believed to be a driving force behind ROS formation, inflammatory cytokine production and the progression to tissue fibrosis [45]. Castellano and colleagues reported a C1INH dependent reduction in recruitment of infiltrating CD163+ monocytes and SMC3a+ dendritic cells in a swine model of renal ischemia reperfusion injury [19]. In our study, we extended the analysis period and performed a kinetic assessment of neutrophils and macrophage infiltration into ischemic grafts to the first 7 days after IR injury. We documented an increase in neutrophil levels that peaked in the first 24 hours decreasing gradually over time at day 3 and 7 post injury and macrophage levels which progressively increased throughout the first 7 days after reperfusion. Interestingly, C1INH attenuated recruitment of both neutrophils and macrophages to injured renal tissue at all analyzed time points. This phenomenon may be partially explained by the reduction in CXCL1 expression and KC release in mice receiving C1INH, which are well-characterized mechanisms of neutrophil recruitment and activation at the site of tissue injury [46]. These data are consistent with published reports in which use of C1INH has been associated with lower circulating IL-8 (KC) levels, reduced of CXCL-1 expression and blunted neutrophil activation in rodent models of septic shock and in the context of hereditary angioedema in humans [47, 48]. In addition, complement inhibition has been previously associated with decreased neutrophil recruitment in models of liver, myocardial and intestinal IRI in rodents.[22, 49, 50]. Taken together, these observations suggest that protection in the setting of IRI by C1INH may be afforded by limited neutrophil migration secondary to C5a, CXCL1 (KC), MCP-1 and IL-6 suppression in the initial phases of the inflammatory response, which results in dysregulated chemotaxis, migration and amplification of the inflammatory response

To our knowledge, this report is one of the first to show a significant long-term anti-fibrotic effect after C1INH treatment in the context of renal IRI. We extended our analysis to 90-days post injury, but due to the experimental design, we could not assess the level of serum markers indicative of renal function in the IRI + PBS group at this time point. Nevertheless, we documented a marked decrease in staining for markers of fibrosis and observed reduced TGF-β1 expression in kidneys from C1INH treated mice when compared to PBS treated controls. In agreement with these findings, a recent study by Curci et al. using a swine model of renal IRI showed that ischemia led to priming of the complement system and renal fibrosis in an Akt dependent manner within 24 hours of injury. More importantly, in their model C1INH blocked Akt activation in endothelial cells in-vivo and prevented endothelial-to-mesenchymal transition known to play an important role in the progression to tissue fibrosis [51]. Moreover, a recent report by Bao et al. investigating the role of the anaphylatoxins C3a and C5a in renal injury showed that C3a deficiency resulted in significantly reduced renal leukocyte infiltration and a marked reduction in tubulo-interstitial inflammation and the degree of tissue fibrosis [52]. Furthermore, a recent report by Boor and colleagues provides compelling evidence of the pro-fibrotic effect of C5a in a mouse model of unilateral ureteral obstruction and indicates that pro-fibrogenic properties are mediated by the C5aR dependent production of TGF-β1 [53]. These results indicate that down-regulation of signaling via C5aR following complement blockade leads to a reduction in TGF-β1 production at the site of injury, which in turn modulates collagen deposition by fibroblasts and limits fibrotic remodeling in the post-reperfusion phase.

One of the main limitations of this study is that we only evaluated the effect of pre-treatment with C1INH in the prevention of renal reperfusion injury. Although our data provides evidence of a protective effect of therapy when administered prior to injury, it remains unclear whether C1INH will have a similar effect when given after the onset of injury/disease. The objective of the current study was to provide proof of concept; however, due to the limited number of observations in some experimental groups, this statement should be interpreted with caution and further investigation is required to establish its efficacy in clinically relevant models of renal IRI. Another aspect that requires further attention is the mechanism behind the anti-fibrotic effect of C1INH therapy. Although we observed an association between complement blockade and reduction in TGF-β1 production, it remains unclear whether this is the result of attenuation of the inflammatory response or more importantly, if C1INH possesses intrinsic anti-fibrotic properties that have yet to be described.

In conclusion, we have demonstrated a protective effect mediated by C1INH in the prevention of acute kidney injury after ischemic insult. Pre-treatment with C1INH prior to induction of IRI inhibited complement activation, prevented C5a cleavage and down-regulated the subsequent inflammatory response. As a result, the progression to fibrosis after ischemia was likely limited by the reduction in the degree of the initial inflammatory injury. This study provides further evidence for the central role of complement in the transition from inflammatory injury to maladaptive tissue repair and highlights the importance of early targeted therapy in the prevention of tubulo-interstitial fibrosis. Our results provide support for C1INH as an attractive therapeutic option for the management of acute and chronic ischemic renal conditions predisposing to kidney fibrosis.

## Supporting information

S1 Fig (A) Creatinine level at 90 days post injury. Note that the IRI+ PBS group did not undergo contralateral nephrectomy, so creatinine values may not be representative of actual renal function at this time point. (B) mRNA expression of IL-4 in renal tissue at 24 and 72 hours post-injury. Expression was normalized to baseline expression of native controls and GAPDH was used as the endogenous control. Data are mean ± SD. Statistical comparison was performed by Kruskal-Wallis and Dunn’s post-hoc correction. *p<0.05, **p<0.01, ***p<0.001.
(PDF)
