# Supplementary figures and images for: Complement inhibition attenuates acute kidney injury after ischemia-reperfusion and limits progression to renal fibrosis in mice
Source: PLoS One. 2017 Aug 23;12(8):e0183701. doi: 10.1371/journal.pone.0183701 (PMC5568291; doi:10.1371/journal.pone.0183701)

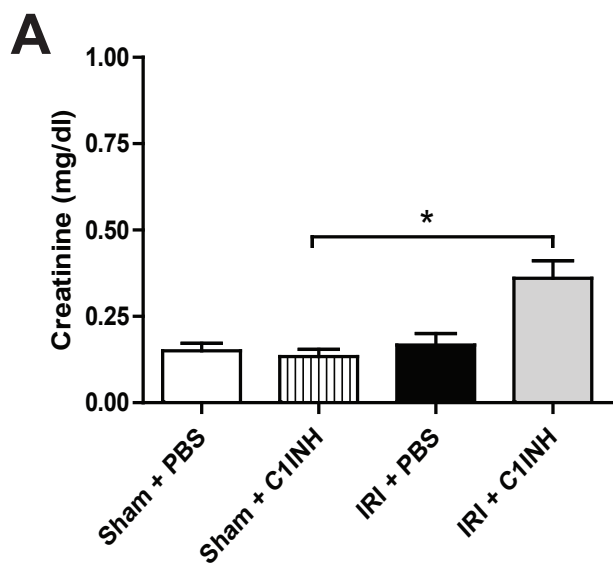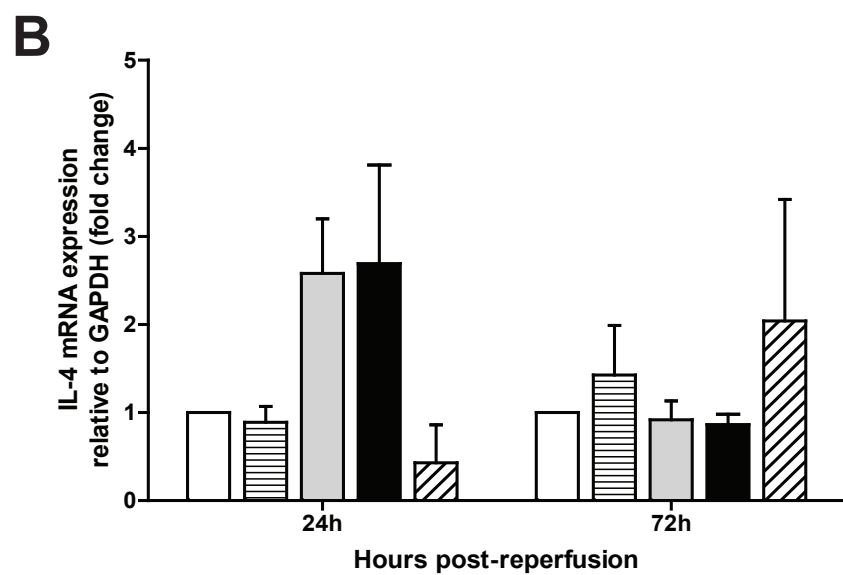

Supplement: S1 Fig — (A) Creatinine level at 90 days post injury. Note that the IRI+ PBS group did not undergo contralateral nephrectomy, so creatinine values may not be representative of actual renal function at this time point. (B) mRNA expression of IL-4 in renal tissue at 24 and 72 hours post-injury. Expression was normalized to baseline expression of native controls and GAPDH was used as the endogenous control. Data are mean ± SD. Statistical comparison was performed by Kruskal-Wallis and Dunn’s post-hoc correction. *p<0.05, **p<0.01, ***p<0.001. (PDF) [file pone.0183701.s001.pdf]
